# Prognostic Value of Heterogeneity Index Derived from Baseline ^18^F-FDG PET/CT in Mantle Cell Lymphoma

**DOI:** 10.3389/fonc.2022.862473

**Published:** 2022-04-14

**Authors:** Fei Liu, Bingxin Gu, Nan Li, Herong Pan, Wen Chen, Ying Qiao, Shaoli Song, Xiaosheng Liu

**Affiliations:** ^1^ Department of Nuclear Medicine, Fudan University Shanghai Cancer Center, Shanghai, China; ^2^ Department of Oncology, Shanghai Medical College, Fudan University, Shanghai, China; ^3^ Center for Biomedical Imaging, Fudan University, Shanghai, China; ^4^ Shanghai Engineering Research Center of Molecular Imaging Probes , Shanghai, China; ^5^ Key Laboratory of Nuclear Physics and Ion-beam Application (MOE), Fudan University, Shanghai, China

**Keywords:** heterogeneity index, mantle cell lymphoma, PET/CT, progression-free survival, ROC

## Abstract

**Objectives:**

Mantle cell lymphoma (MCL) represents a group of highly heterogeneous tumors, leading to a poor prognosis. Early prognosis prediction may guide the choice of therapeutic regimen. Thus, the purpose of this study was to investigate the potential application value of heterogeneity index (HI) in predicting the prognosis of MCL.

**Methods:**

A total of 83 patients with histologically proven MCL who underwent baseline fluorine-18-fluorodeoxyglucose positron emission tomography/computed tomography (^18^F-FDG PET/CT) were retrospectively enrolled. The clinicopathologic index and PET/CT metabolic parameters containing maximum and mean standard uptake value (SUV_max_ and SUV_mean_), metabolic tumor volume (MTV), total lesion glycolysis (TLG), and HI were evaluated. Receiver operating characteristic (ROC) curve analyses were performed to determine the optimal cutoff values of the parameters for progression-free survival (PFS) and overall survival (OS). Univariate and multivariate Cox regression were used to assess relationships between risk factors and recurrence. Kaplan–Meier plots were applied for survival analyses.

**Results:**

In univariate analyses, age [HR = 2.51, 95% CI = 1.20–5.24, *p* = 0.041 for body weight (BW)] and HI-BW (HR = 4.17, 95% CI = 1.00–17.38, *p* = 0.050) were significantly correlated with PFS. In multivariate analyses, age (HR = 2.61, 95% CI = 1.25–5.47, *p* = 0.011 for BW) and HI-BW (HR = 4.41, 95% CI = 1.06–18.41, *p* = 0.042) were independent predictors for PFS, but not for OS. B symptoms (HR = 5.00, 95% CI = 1.16–21.65, *p* = 0.031 for BW) were an independent prognostic factor for OS, but not for PFS. The other clinicopathologic index and PET/CT metabolic parameters were not related to outcome survival in MCL.

**Conclusion:**

The age and HI derived from baseline PET/CT parameters were significantly correlated with PFS in MCL patients.

## Introduction

Mantle cell lymphoma (MCL) is a group of invasive small B-cell lymphoma derived from primary and secondary lymphoid follicle mantle lymphocytes, accounting for 3% to 10% of all non-Hodgkin lymphoma (NHL) (1). According to the 2016 WHO classification, MCL is divided into classical, inert leukemic non-nodal, and *in situ* mantle cell tumor subtypes (2, 3). As most patients with MCL are diagnosed at advanced stage (Ann Arbor III–IV), the prognosis is very poor (4). Thus, a comprehensive assessment before treatment may benefit patients with MCL (5).

MCL international prognostic index (MIPI) score is usually used in the prognosis evaluation of MCL, which classifies the patients into low-risk, medium-risk, and high-risk groups (6). In 2008, a combined biologic index (MIPI-b), integrating MIPI and the Ki-67 index, was established as a prognostic tool for treatment response, recurrence, and survival prediction of MCL (7). In 2016, Hoster et al. demonstrated that a modified combination of Ki-67 index and MIPI (MIPI-c) could further divide MCL patients into four groups with different prognosis (8). Other biologic markers (e.g., p53 and microRNAs) have also been combined with MIPI as the prognostic tools (9, 10). Although these biologic markers are promising, they could not adequately be consistent with the heterogeneity of clinical outcomes. Herein, it is urgent to find more comprehensive prognostic factors of MCL (8).

Fluorine-18-fluorodeoxyglucose positron emission tomography/computed tomography (^18^F-FDG PET/CT) is a recommended and useful tool to evaluate the prognosis and treatment response of MCL (4). Bailly et al. (11) demonstrated a significant correlation between maximum standard uptake value (SUV_max_) with progression-free survival (PFS) and overall survival (OS) using a threshold value of 10.3, while no significant correlation was observed with a threshold value of 4.7. However, Bodet-Milin et al. (12) and Hosein et al. (13) reported no significant association between SUV_max_ with PFS or OS using a threshold value of 6.0. Furthermore, metabolic tumor volume (MTV) was considered significant with PFS and OS only for univariate analyses. Moreover, another study reported a significant difference in PFS and OS with MTV and total lesion glycolysis (TLG) (14).

The above results showed that the potential role of the parameters in MCL prognostication were controversial and no shared prognostic indexes to identify this subset of NHL are available currently. The reason may be related to the heterogeneity of MCL. At present, MCL is considered to be a group of tumors with strong heterogeneity in biology, morphology, immunophenotype, and clinical process (15–17). The heterogeneity index (HI) of SUV measured by PET/CT is a potential index that is associated with tumor heterogeneity (18–21). Gong et al. showed that HI measured at baseline ^18^F-FDG PET/CT was a potential predicator for first-line treatment outcome in triple-negative breast cancer patients (21). Lee et al. revealed that HI may be a useful prognostic marker in uterine leiomyosarcoma (19). However, there are few heterogeneity-related factors that have been investigated in MCL. In this study, we aim to investigate the relationship between survival outcome with clinicopathologic and PET/CT parameters, and to verify the potential application of HI derived from PET/CT in predicting the prognosis of MCL.

## Materials and Methods

### Patients

**Figure 1:**
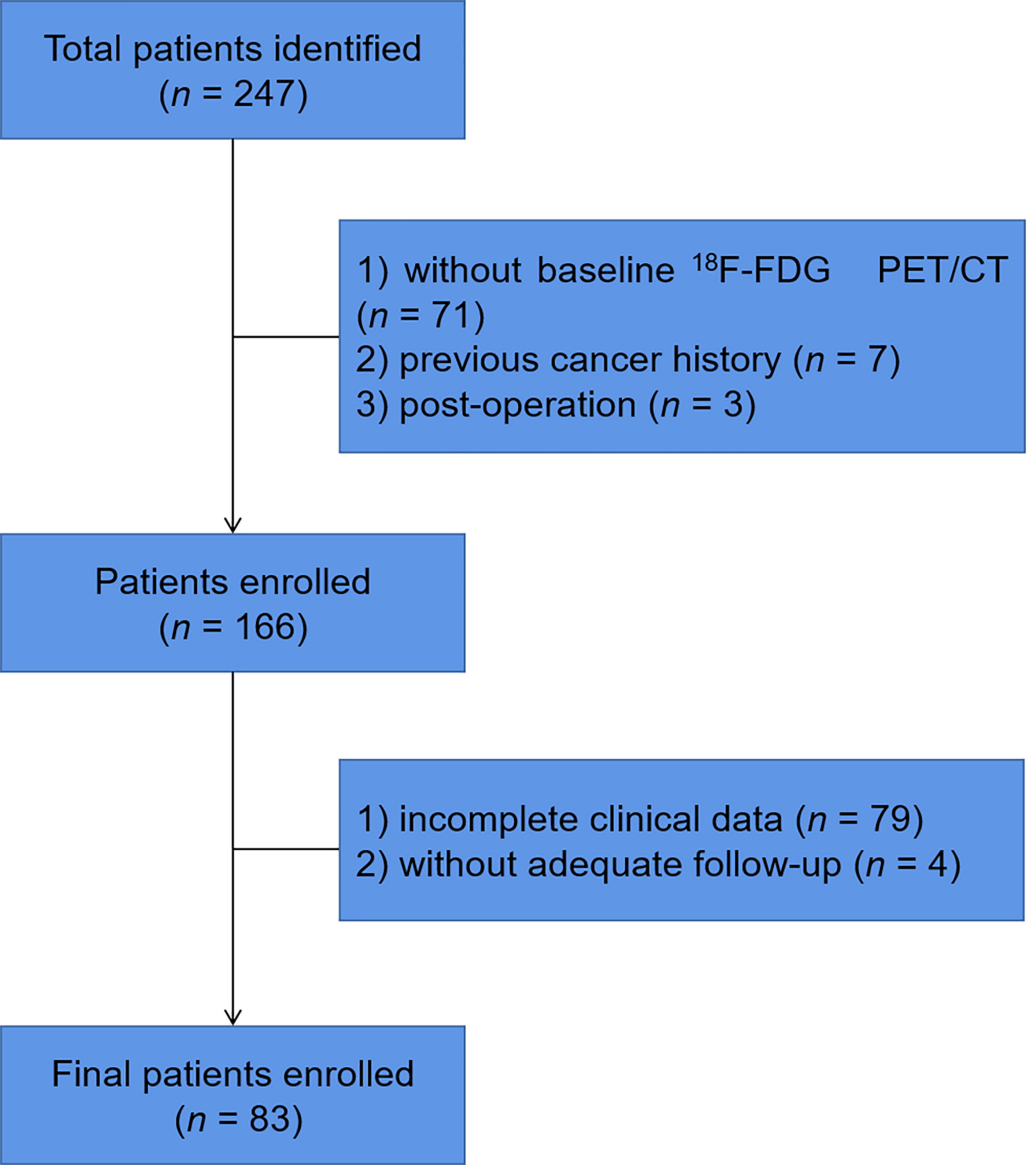
Flow chart of patient selection and exclusion.

We retrospectively identified MCL patients with pathological results who had undergone baseline ^18^F-FDG PET/CT from Fudan University Shanghai Cancer Center (FUSCC) between January 2011 and December 2020. Patients were excluded if they had no baseline ^18^F-FDG PET/CT, had a previous cancer history, had undergone surgery before ^18^F-FDG PET/CT scan, or had incomplete clinical data or follow-up (**Figure 1**). All patients were treated according to the latest Chinese Lymphoma Guidelines Consensus. The study was approved by the Ethics Committee of FUSCC and informed consents were waived.

A total of 83 patients with histologically proven MCL were recruited into this study. We reviewed the medical records of these patients and the following clinical, laboratory, and biological features: age, sex, body mass index (BMI), Ann Arbor stage, B symptoms, bulky disease, splenomegaly, lactate dehydrogenase (LDH), β2-microglobulin levels, MIPI score, Ki-67 proliferation index, and metabolic features derived from the baseline ^18^F-FDG PET/CT scans. The LDH and β2-microglobulin levels were divided into two groups with the cutoff value of 245 U/L and 2.8 mg/L, respectively. Bulky disease was defined with CT when the mass was ≥10 cm, while splenomegaly was defined when the maximum length diameter of spleen was >13 cm (22). The patients were divided into high Ki-67 (>20%) and low Ki-67 (≤20%) groups according to the Ki-67 expression level. Patients with a MIPI score higher than 2 were classified in the high MIPI score group.

### ^18^F-FDG PET/CT Acquisition and Reconstruction Parameters

All the patients underwent baseline ^18^F-FDG PET/CT imaging using a Siemens biograph PET/CT scanner (Knoxville, Tennessee, USA). The patients were fasted for at least 6 h and the blood glucose was maintained below 10 mmol/L before the examination. Each patient was intravenously injected with ^18^F-FDG at a dose of 3.7 MBq/kg and continued to rest for approximately 60 min before a PET/CT scan from the head to the mid-thigh was performed. Before PET scanning, a CT scan was performed with the following parameters: 120 kV, 80–250 mA, slice thickness 5 mm, and 0.5 s per rotation. PET images were reconstructed using a three-dimensional ordered subsets expectation maximum (OSEM) algorithm with CT for attenuation.

### Imaging Interpretation

PET and CT scans were fused and reviewed using the Fusion Viewer software from the manufacturer. The PET/CT images were read by two experienced nuclear medicine physicians blinded to the prior reports. For quantitative analyses, region of interest (ROI) was drawn using Syngo.via software (Siemens) over the regions of tumors. The SUV_max_ and mean standard uptake value (SUV_mean_) adjusted to body weight (BW), lean body mass (LBM), and body surface area (BSA) were automatically generated. MTV was calculated automatically according to the threshold of 41% SUV_max_ in hypermetabolic regions, and TLG was calculated as SUV_mean_ × MTV (23). HI was calculated as dividing SUV_max_ by SUV_mean_ to evaluate the heterogeneity of tumors (24).

### Statistical Analyses

All statistical analyses were carried out using SPSS software (version 26.0, IBM, New York, USA). Receiver operating characteristic (ROC) curve analyses for PFS and OS of 5 years were performed to determine the optimal cutoff values of the parameters, and parameters with an area under the ROC curve (AUC) greater than 0.5 were retained for further analyses. PFS was defined from the date of baseline ^18^F-FDG to the first disease progression, recurrence, or death (months), and progression/recurrence was considered when the number or dimension of previous lesion increased or new lesion appeared. OS was defined from the date of baseline PET/CT scan until the time of death due to any cause, or the date of last follow-up. Univariate and multivariate Cox regression were used to determine factors in relation to PFS and OS, and variables with a *p*-value of less than 0.1 in the univariate analyses were included in the further multivariate analyses. Kaplan–Meier plots were performed for survival curves, and the log-rank test were used to determine differences between two curves. A *p*-value of less than 0.05 was defined as statistically significant.

## Results

### Patient Characteristics

The characteristics of the 83 MCL patients (64 men and 19 women; average age, 59.76 years; range 43–76 years) are presented in **Table 1** and **Supplementary Table S1**. The average BMI was 23.20 kg/m^2^ with a range of 16.02–30.80 kg/m^2^. All patients were staged according to the Ann Arbor system, of whom 1% were stage I, 6% stage II, 22% stage III, and 71% stage IV. Of the 83 patients, 13 (16%) had B symptoms, 19 (23%) had bulky disease, and 27 (33%) had splenomegaly status. High LDH and β2-microglobulin levels were presented in 11 (13%) and 25 (30%) patients, respectively. Fifty-two (63%) patients had high MIPI score of >2 and high Ki-67 score was available for 64 (77%) patients.

**Table 1:** Baseline characteristics of patients (*n* = 83).

Characteristic	Patients, *n* (%)	Average (range)
Age (years)		59.76 (43–76)
Sex		
Male	64 (77%)	
Female	19 (23%)	
BMI (kg/m^2^)		23.20 (16.02–30.80)
Ann Arbor		
I	1 (1%)	
II	5 (6%)	
III	18 (22%)	
IV	59 (71%)	
B symptoms	13 (16%)	
Bulky disease	19 (23%)	
Splenomegaly	27 (33%)	
LDH (U/L)		
≤245	72 (87%)	
>245	11 (13%)	
β2-microglobulin (mg/L)		
≤2.8	58 (70%)	
>2.8	25 (30%)	
MIPI score		
Low (≤2)	31 (37%)	
High (>2)	52 (63%)	
Ki-67 score		
≤20%	19 (23%)	
>20%	64 (77%)	
SUV_max_-BW		9.08 (0.35–38.22)
SUV_mean_-BW		3.66 (0.21–14.66)
MTV		614.31 (5.61–3,675.55)
TLG-BW		2510.42 (9.47–13,811.40)
HI-BW		2.84 (1.50–29.63)

LDH, lactate dehydrogenase; MIPI, international prognostic index; SUV_max_, maximal standard uptake value; SUV_mean_, mean standard uptake value; HI, heterogeneity index; BW, body weight; MTV, metabolic tumor volume; TLG, total lesion glycolysis.

All patients underwent a baseline PET/CT scan and FDG avidity was identified in 100% of the MCL patients. PET/CT parameters adjusted to BW, SUV_max_-BW, and SUV_mean_-BW were 9.08 (range 0.35–38.22) and 3.66 (range 0.21–14.66), and the MTV, TLG-BW, and HI-BW were 614.31 (range 5.61–3,675.55), 2,510.42 (range 9.47–13,811.40), and 2.84 (range 1.50–29.63) (**Table 1**). For the parameters adjusted to LBM, the SUV_max_-LBM and SUV_mean_-LBM were 7.13 (range 0.37–29.88) and 2.91 (range 0.23–11.98), and the TLG-LBM and HI-LBM were 2003.46 (range 7.44–11,487.70) and 2.51 (range 1.49–5.54). For the parameters adjusted to BSA, the SUV_max_-BSA and SUV_mean_-BSA were 2.43 (range 0.33–9.76) and 1.01 (range 0.20–3.92), and the TLG-BSA and HI-BSA were 682.52 (range 2.43–4,073.61) and 2.50 (range 0.73–5.54) (**Supplementary Table S1**).

### ROC Curve Analyses of Prognostic Factors

Of the 83 patients, 35 (42%) had disease progression/recurrence at a median of 20.06 months, and death occurred in 8 (10%) patients at a median of 31.50 months. The ROC survival curve analyses for PFS and OS are presented in **Table 2** and **Supplementary Table S2**. The study used the 5-year PFS and OS rates as the main study end point, making ROC curves for the clinical and metabolic factors, and the maximum cross-sectional AUCs were defined as the optimal cutoff values.

**Table 2:** ROC curve analyses of prognostic factors for PFS and OS in MCL.

Parameter	PFS			OS		
	Cutoff	AUC (95% CI)	*p*-value	Cutoff	AUC (95% CI)	*p*-value
Age	58.50	0.58 (0.45–0.70)	**0.011**	60.50	0.65 (0.47–0.83)	0.165
BMI	23.10	0.49 (0.36–0.61)	0.504	19.57	0.30 (0.13–0.47)	0.060
SUV_max_-BW	5.90	0.48 (0.35–0.60)	0.343	7.60	0.57 (0.35–0.79)	0.517
SUV_mean_-BW	1.89	0.46 (0.33–0.58)	0.248	1.99	0.52 (0.30–0.74)	0.841
MTV	31.96	0.46 (0.33–0.59)	0.218	253.72	0.57 (0.39–0.76)	0.497
TLG-BW	70.08	0.47 (0.34–0.59)	0.217	642.81	0.62 (0.44–0.81)	0.254
HI-BW	1.94	0.57 (0.45–0.70)	**0.032**	2.45	0.55 (0.32–0.78)	0.632

ROC, receiver operating characteristic; PFS, progression-free survival; OS, overall survival; AUC, area under the curve; CI, confidence intervals.

Results with a p-value of <0.05 were considered significant and were bolded.

As shown in **Table 2**, considering PFS, the optimal cutoff values of age and HI-BW were 58.5 years and 1.94, the AUCs were 0.58 (range 0.45–0.70) and 0.57 (range 0.45–0.70), and the *p*-values were 0.011 and 0.032, respectively. We performed SPSS analyses with LBM- and BSA-related SUV parameters, with similar results (**Supplementary Table S2**). For HI-LBM and HI-BSA, the optimal cutoff values were 1.98 and 1.94, the AUCs were 0.57 (range 0.45–0.7) and 0.59 (range 0.47–0.71), and the *p*-values were 0.041 and 0.029 for PFS.

### Univariate and Multivariate Survival Analyses in Relation to PFS and OS

Taking the 5-year PFS and OS as the end points, the clinical and metabolic factors were included into the univariate Cox survival analyses, as shown in **Table 3**, and age over 58.50 years and high HI-BW were significantly related to PFS (*p* = 0.041 and 0.050), but not with OS (*p* = 0.128 and 0.253). B symptoms were significantly related to OS (*p* = 0.004), but not with PFS (*p* = 0.780). Similarly, for LBM- and BSA-related SUV parameters, high HI-BLM and HI-BSA showed significance with PFS (*p* = 0.055 and 0.046), but not with OS (*p* = 0.253 and 0.596, **Supplementary Table S3, S4**). However, SUV_max_, SUV_mean_, MTV, and TLG were not significantly correlated with both PFS and OS (**Table 3** and **Supplementary Tables S3, S4**).

**Table 3:** Univariate and multivariate analyses of BW-related prognostic factors in relation to PFS and OS using the Cox regression model.

	Univariate analysis		Multivariate analysis	
	HR (95% CI)	*p*-value	HR (95% CI)	*p*-value
**PFS**				
Age	2.51 (1.20–5.24)	0.041	2.61 (1.25–5.47)	**0.011**
Sex	1.96 (0.83–4.63)	0.125		
BMI	1.25 (0.64–2.45)	0.508		
B symptoms	0.86 (0.30–2.46)	0.780		
Bulky disease	0.75 (0.31–1.80)	0.514		
Splenomegaly	0.56 (0.25–1.24)	0.151		
LDH	0.59 (0.18–1.92)	0.376		
β2-microglobulin	0.90 (0.42–1.93)	0.786		
MIPI score	1.73 (0.84–3.54)	0.135		
Ki-67 score	1.74 (0.72–4.21)	0.217		
SUV_max_-BW	1.49 (0.65–3.41)	0.350		
SUV_mean_-BW	22.88 (0.007–80195.58)	0.452		
MTV	3.24 (0.44–23.78)	0.248		
TLG-BW	3.25 (0.44–23.85)	0.247		
HI-BW	4.17 (1.00–17.38)	0.050	4.41 (1.06–18.41)	**0.042**
Ann Arbor	–	0.758		
I	Reference			
II	1361.22 (0–5.70 × 10^88^)	0.943		
III	3979.67 (0–1.65 × 10^89^)	0.934		
IV	2982.50 (0–1.24 × 10^89^)	0.937		
**OS**				
Age	3.47 (0.70–17.23)	0.128		
Sex	3.12 (0.38–25.81)	0.290		
BMI	21.17 (0–8.95 × 10^10^)	0.787		
B symptoms	7.75 (1.90–31.56)	0.004	5.00 (1.16–21.65)	**0.031**
Bulky disease	1.41 (0.28–7.05)	0.673		
Splenomegaly	0.77 (0.15–3.82)	0.747		
LDH	2.56 (0.51–12.79)	0.252		
β2-microglobulin	3.60 (0.85–15.21)	0.082	1.88 (0.39–0.17)	0.435
MIPI score	2.13 (0.43–10.58)	0.355		
Ki-67 score	2.44 (0.30–20.22)	0.407		
SUV_max_-BW	3.68 (0.73–18.50)	0.114		
SUV_mean_-BW	24.08 (0.001–1.12 × 10^6^)	0.562		
MTV	6.26 (0.74–52.85)	0.092	1.33 (0.04–46.40)	0.876
TLG-BW	5.91 (0.71–48.96)	0.099	2.76 (0.10–80.24)	0.554
HI-BW	2.30 (0.55–9.65)	0.253		
Ann Arbor	–	0.938		
I	Reference			
II	0.99 (0–2.16 × 10^189^)	1.000		
III	737.45 (0–1.57 × 10^188^)	0.976		
IV	1432.18 (0–3.03 × 10^188^)	0.973		

HR, hazard ratio.

Results with a p-value of <0.05 were considered significant and were bolded.

**Figure 2:**
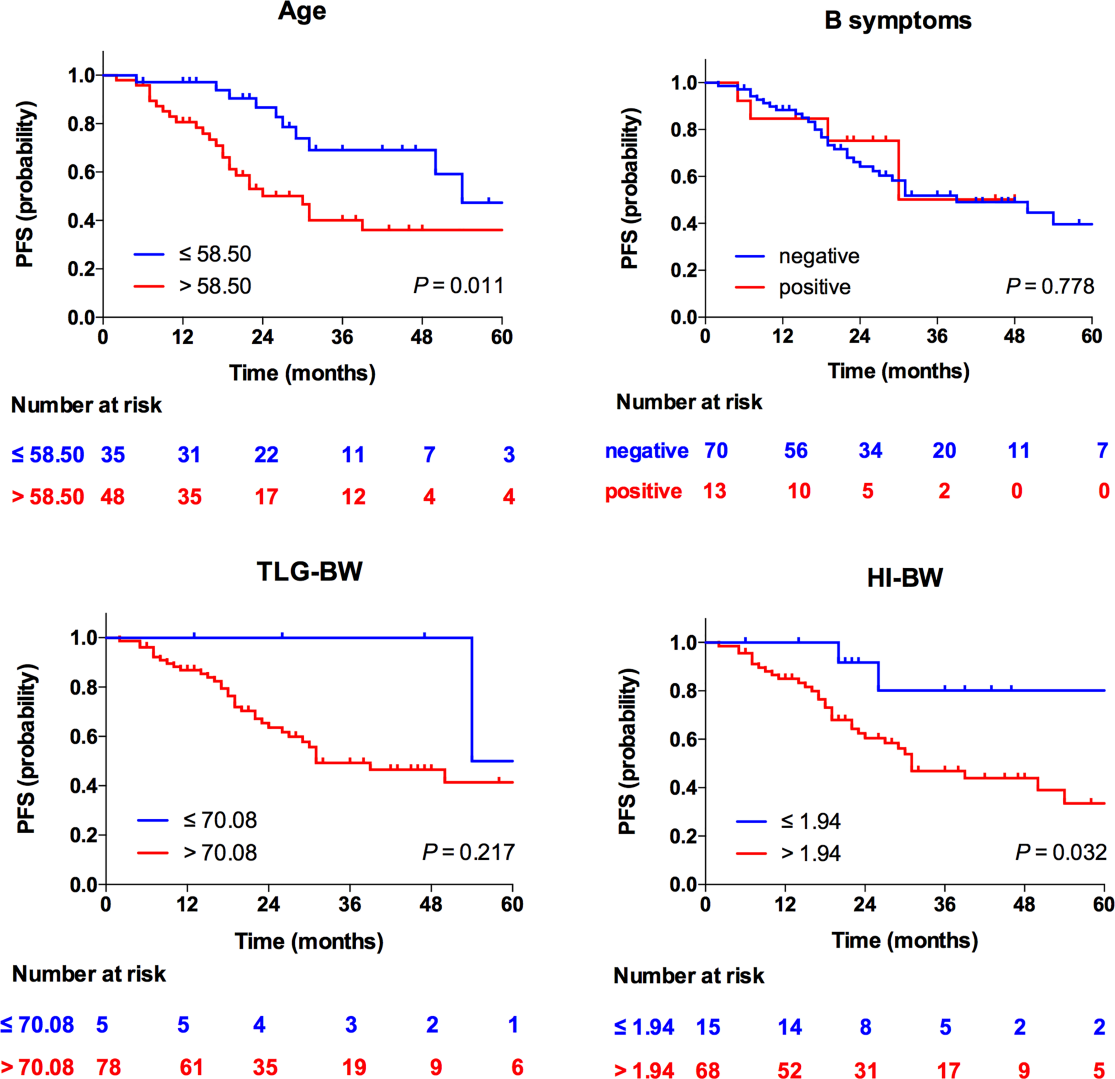
Kaplan–Meier curves for PFS according to age, B symptoms, TLG-BW, and HI-BW.

**Figure 3:**
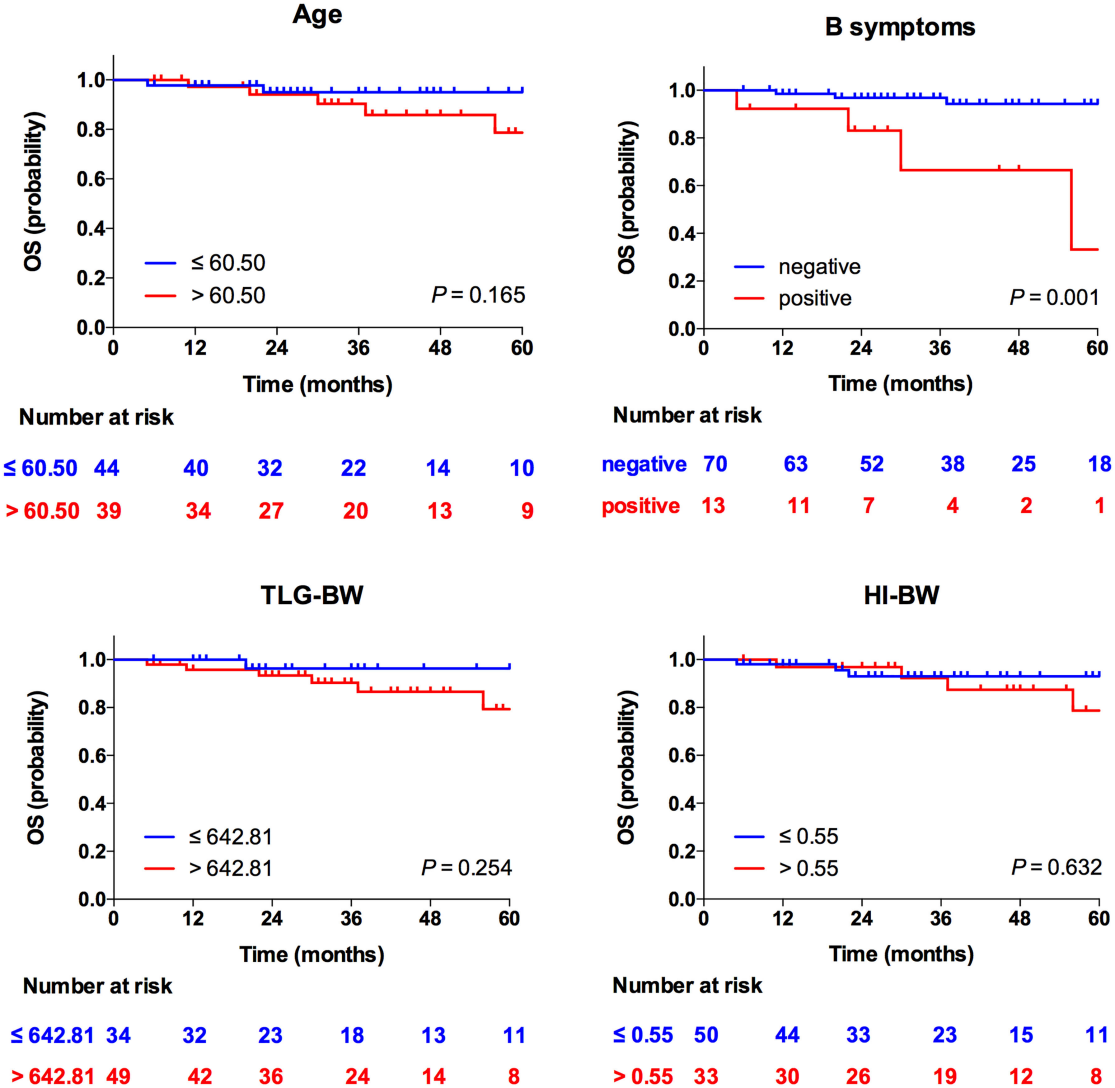
Kaplan–Meier curves for OS according to age, B symptoms, TLG-BW, and HI-BW.

**Figure 4:**
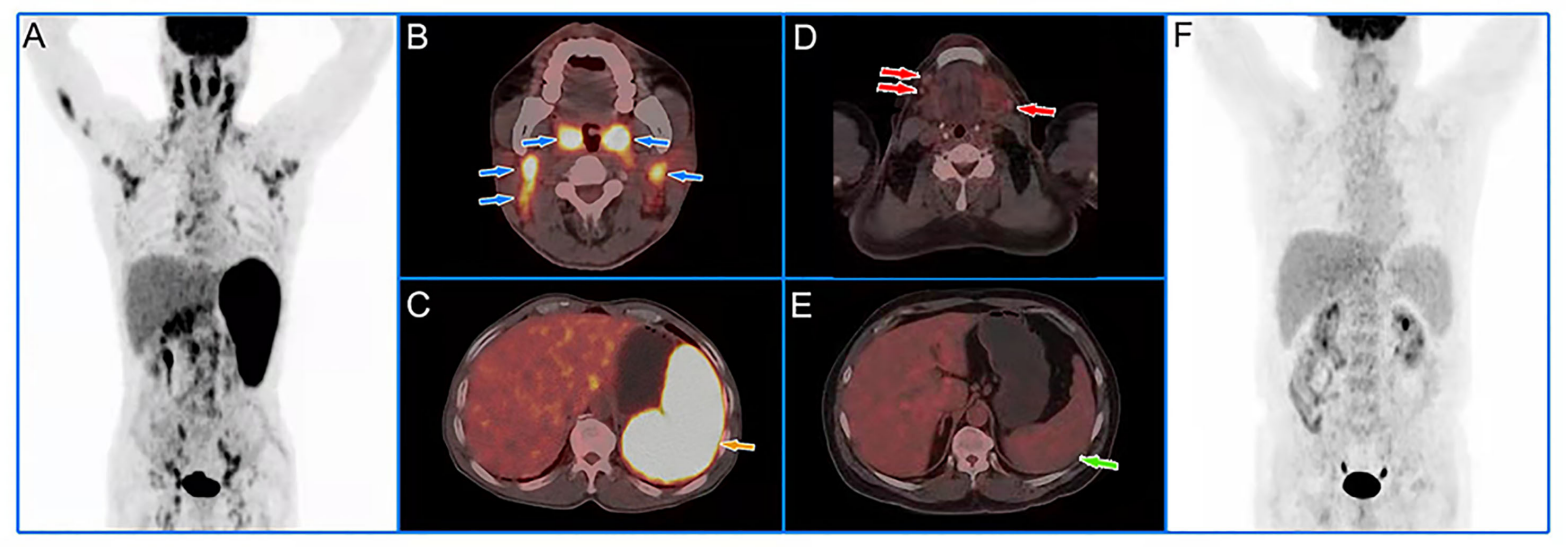
Representative cases of baseline ^18^F-FDG PET/CT images. A 60-year-old male patient with low HI had extensive lymph node lymphoma invasion and lymphoma infiltration into the oropharynx and spleen **(A)**. The blue and yellow arrows represented the oropharynx, cervical lymph nodes **(B)**, and spleen **(C)**, respectively. The lesions had an SUV_max_ of 11.09, an SUV_mean_ of 7.10, and a HI of 1.56. We followed up for 39 months and the patient had no recurrence. A 55-year-old male patient with high HI had extensive critical-sized lymph node lymphoma invasion and no lymphoma infiltration into the organs **(F)**. The red and green arrows represented the submaxillary lymph nodes **(D)** and spleen **(E)**, respectively. The lesions had an SUV_max_ of 3.35, an SUV_mean_ of 1.13, and a HI of 2.96. The patient developed local recurrence after 29 months of follow-up.

At multivariate analyses, patients with old age (>58.50 years) had a significantly shorter PFS (30 months versus 54 months), and the PFS of patients with high HI (>1.94 for HI-BW and HI-BSA, >1.98 for HI-LBM) was significantly shorter than that of patients with low HI (31 months versus not-reached for HI-BW, HI-LBM, and HI-BSA). Age (HR = 2.61, 95% CI = 1.25–5.47, *p* = 0.011 for BW; HR = 2.66, 95% CI = 1.27–5.58, *p* = 0.010 for LBM; HR = 2.63, 95% CI = 1.26–5.50, *p* = 0.010 for BSA), HI-BW (HR = 4.41, 95% CI = 1.06–18.41, *p* = 0.042), HI-LBM (HR = 3.46, 95% CI = 1.05–11.33, *p* = 0.041), and HI-BSA (HR = 4.54, 95% CI = 1.09–18.96, *p* = 0.038) were proved to be correlated prognostic factors for PFS. B symptoms showed significant correlation with OS (HR = 5.00, 95% CI = 1.16–21.65, *p* = 0.031 for BM; HR = 9.04, 95% CI = 1.89–43.16, *p* = 0.006 for LBM; HR = 4.97, 95% CI = 1.15–21.52, *p* = 0.032 for BSA), but were not significantly correlated with prognosis considering PFS (**Table 3** and **Supplementary Tables S3, S4**). Kaplan–Meier PFS and OS curves of the parameters are displayed in **Figures 2**, **3** and **Supplementary Figure S1**. Representative images of ^18^F-FDG with high and low HI are shown in **Figure 4**.

## Discussion

MCL has the characteristics of both invasive and inert lymphoma, and the prognosis of MCL is very poor. Despite the more aggressive treatment, the progression/recurrence of the disease is still frequent; accurate and effective treatment is very important for MCL patients (25, 26). Research showed that the heterogeneity of MCL leads to different treatment effects. How to identify the heterogeneity of MCL and its corresponding clinical characteristics in order to seek more individualized treatment is a long-term research goal (17, 27, 28). HI, the SUV_max_/SUV_mean_ ratio, which is calculated from PET/CT images parameters, has been investigated in tumors recently. A previous study has showed that baseline ^18^F-FDG HI could be used to predict the survival rate of patients with advanced nasopharyngeal carcinoma (29) and that the HI derived from ^18^F-fluoroestradiol (^18^F-FES) PET/CT could reflect the estrogen receptor (ER) expression in breast cancer patients (30). To the best of our knowledge, our study is the first research that demonstrated a significant correlation of HI with MCL PFS prognosis, which may further guide the clinical treatment decision-making of MCL patients and benefit the patients to the maximum extent. SUV has traditionally been used to define glucose metabolic activity in PET/CT imaging, and when calculating SUV, BW is usually used as body size measurement; however, some researchers may prefer LBM or BSA to measure body size (31). Our study showed that the prognostic value of HI in MCL patients did not differ in the above three measurement modes. However, in our study, HI showed no significant difference for OS; this might be due to the fact that the number of death events was only 8, and our findings might be inaccurate due to the small sample size.

MIPI score is the most commonly used model in MCL, which combines Eastern Cooperative Oncology Group (ECOG) performance status, age, leukocyte count, and lactate dehydrogenase (6). The 2018 British Society for Haematology (BSH) MCL guidelines point out that the patients’ age, complications, PS scores, and treatment objectives are the factors to be considered before treatment. For patients aged ≤65 years or generally in good condition and suitable for autologous stem cell transplantation (ASCT), induction therapy with high-dose cytarabine should be selected, ASCT consolidation should be performed after remission, and treatment with rituximab can be further beneficial. For patients aged >65 years or generally in poor condition and unsuitable for ASCT, immunochemotherapy with less adverse reactions and better tolerance should be selected (32). In our study, age was an independent prognostic factor for PFS in MCL patients, which was consistent with the guidelines. MCL patients can cause systemic manifestations in the body; patients who have any of the following symptoms are defined as B symptoms positive: unexplained fever (often over 38.0°C), weight loss of more than 10% within 6 months, and night sweats. The presence of B symptoms usually predicts a poor prognosis (33). In our research, B symptoms were an independent prognostic factor for OS.

SUV_max_, SUV_mean_, MTV, and TLG are common PET/CT parameters used in clinical research. Tsukamoto et al. stated that SUV_max_ ≤ 6.5 had significant correlation with PFS in patients with relapsed indolent lymphoma treated with ^90^Y-ibritumomab tiuxetan (34). Feng et al. indicated that baseline SUV_max_ measured on ^18^F-FDG in T-cell lymphoblastic lymphoma was significantly related to PFS and OS (35). Okuyucu et al. demonstrated that SUV_mean_ was a potential risk factor for OS in primary extranodal lymphoma (36). Instead, in our research, SUV_max_ and SUV_mean_ showed no significance with PFS or OS in MCL patients. MTV and TLG reflect the volume and total glycolysis of metabolically active tumors, respectively, and MTV and TLG have been proven to be useful indicators to measure tumor invasiveness and predict the treatment response of tumors (37). Albano et al. demonstrated that baseline MTV and TLG were significantly correlated with PFS in MCL patients (14); however, in our study, the metabolic tumor features (MTV and TLG) showed no significant relationship with both PFS and OS. Previous studies showed similar results; Mayerhoefer et al. demonstrated that TLG was an independent prognostic factor of 2-year PFS in mucosa-associated lymphoid tissue (MALT) lymphoma treated with CD20-antibody-based immunotherapy, but another study claimed that the PET/CT parameters (MTV and TLG) were not related to PFS or OS in MALT lymphoma (38, 39). The possible reason for the results may arise from the heterogeneity of patients recruited.

However, this study still has some limitations. Firstly, the number of patients recruited was low and the interim/post-treatment response evaluation using ^18^F-FDG PET/CT was absent, which needs to be further validated. Secondly, the number of death events was small, and our findings on OS prognosis might be inaccurate. Thirdly, the results also lack further confirmation by multicenter and prospective studies in MCL patients.

## Conclusion

In conclusion, our findings showed that age and HI derived from PET/CT metabolic factors were helpful independent prognostic factors to predict long-term PFS in MCL patients. However, larger-scale clinical studies are needed to better verify the prognostic role of HI in MCL patients.

## Data Availability Statement

The original contributions presented in the study are included in the article/**Supplementary Material**. Further inquiries can be directed to the corresponding author.

## Ethics Statement

Written informed consent was not obtained from the individual(s) for the publication of any potentially identifiable images or data included in this article.

## Author Contributions

FL and BG designed the studies. FL wrote the manuscript. NL, HP, WC, and YQ gathered the data. FL and BG analyzed the data. SS and XL participated in revising the manuscript. All authors contributed to the article and approved the submitted version.

## Funding

This work was funded by the National Natural Science Foundation of China (Grant number 82102097, 81771861, 81971648, and 81901778), the Talent Introduction Fund of Fudan University Shanghai Cancer Center (Grant number YJRC202104), the National Key Research and Development Program of China (Grant number 2020YFA0909000), and Shanghai Anticancer Association Program (Grant number HYXH2021004).

## Conflict of Interest

The authors declare that the research was conducted in the absence of any commercial or financial relationships that could be construed as a potential conflict of interest

## Publisher’s Note

All claims expressed in this article are solely those of the authors and do not necessarily represent those of their affiliated organizations, or those of the publisher, the editors and the reviewers. Any product that may be evaluated in this article, or claim that may be made by its manufacturer, is not guaranteed or endorsed by the publisher.
