# Supplementary material for: Prognostic Value of Heterogeneity Index Derived from Baseline 18F-FDG PET/CT in Mantle Cell Lymphoma
Source: Front Oncol. 2022 Apr 14;12:862473. doi: 10.3389/fonc.2022.862473 (PMC9047855; doi:10.3389/fonc.2022.862473)
Supplement: Supplementary file 1 [file DataSheet_1.docx]

Supplementary Material

**TABLE S1** **|** Baseline characteristics of LBM and BSA-related SUV parameters in patients (*n* = 83).

| Characteristic | Average (range) |
| --- | --- |
| SUV_max_-LBM | 7.13 (0.37-29.88) |
| SUV_mean_-LBM | 2.91 (0.23-11.98) |
| TLG-LBM | 2003.46 (7.44-11487.70) |
| HI-LBM | 2.51 (1.49-5.54) |
| SUV_max_-BSA | 2.43 (0.33-9.76) |
| SUV_mean_-BSA | 1.01 (0.20-3.92) |
| TLG-BSA | 682.52 (2.43-4073.61) |
| HI-BSA | 2.50 (0.73-5.54) |

*SUV_max_* maximal standard uptake value, *SUV_mean_* mean standard uptake value, *HI* heterogeneity index, *LBM* lean body mass, *BSA* body surface area, *TLG* total lesion glycolysis.

**TABLE S2** **|** ROC curve analyses of LBM and BSA-related prognostic factors for PFS and OS in MCL.

| Parameter | PFS | | |  | | OS | | | |
| --- | --- | --- | --- | --- | --- | --- | --- | --- | --- |
|  | Cutoff | AUC (95% CI) | *P* value | |  | | Cutoff | AUC (95% CI) | *P* value |
| SUV_max_-LBM | 3.88 | 0.47 (0.35-0.60) | 0.376 | |  | | 6.00 | 0.59 (0.37-0.80) | 0.431 |
| SUV_mean_-LBM | 1.57 | 0.45 (0.33-0.58) | 0.799 | |  | | 7.82 | 0.55 (0.33-0.77) | 0.677 |
| TLG-LBM | 84.81 | 0.46 (0.33-0.59) | 0.402 | |  | | 520.57 | 0.63 (0.44-0.81) | 0.241 |
| HI-LBM | 1.98 | 0.57 (0.45-0.70) | **0.041** | |  | | 2.45 | 0.56 (0.32-0.79) | 0.611 |
| SUV_max_-BSA | 1.32 | 0.47 (0.35-0.59) | 0.376 | |  | | 2.00 | 0.60 (0.38-0.80) | 0.405 |
| SUV_mean_-BSA | 0.23 | 0.42 (0.30-0.54) | 0.501 | |  | | 2.61 | 0.53 (0.30-0.75) | 0.817 |
| TLG-BSA | 1440.19 | 0.45 (0.33-0.58) | 0.250 | |  | | 169.74 | 0.62 (0.43-0.81) | 0.260 |
| HI-BSA | 1.94 | 0.59 (0.47-0.71) | **0.029** | |  | | 2.45 | 0.56 (0.33-0.79) | 0.568 |

*ROC* receiver operating characteristic, *PFS* progression-free survival, *OS* overall survival, *AUC* area under the curve, *CI* confidence intervals.

Results with a *P* value of < 0.05 were considered significant and were bolded.

**TABLE S3 |** Univariate and multivariate analyses of LBM-related prognostic factors in relation to PFS and OS using the Cox regression model.

|  | Univariate analysis | |  | Multivariate analysis | |
| --- | --- | --- | --- | --- | --- |
|  | HR (95% CI) | *P* value |  | HR (95% CI) | *P* value |
| **PFS** |  |  |  |  |  |
| Age | 2.51 (1.20-5.24) | 0.041 |  | 2.66 (1.27-5.58) | **0.010** |
| Sex | 1.96 (0.83-4.63) | 0.125 |  |  |  |
| BMI | 1.25 (0.64-2.45) | 0.508 |  |  |  |
| B symptoms | 0.86 (0.30-2.46) | 0.780 |  |  |  |
| Bulky disease | 0.75 (0.31-1.80) | 0.514 |  |  |  |
| Splenomegaly | 0.56 (0.25-1.24) | 0.151 |  |  |  |
| LDH | 0.59 (0.18-1.92) | 0.376 |  |  |  |
| β2-microglobulin | 0.90 (0.42-1.93) | 0.786 |  |  |  |
| MIPI score | 1.73 (0.84-3.54) | 0.135 |  |  |  |
| Ki-67 score | 1.74 (0.72-4.21) | 0.217 |  |  |  |
| MTV | 3.24 (0.44-23.78) | 0.248 |  |  |  |
| SUV_max_-LBM | 1.89 (0.45-7.85) | 0.387 |  |  |  |
| SUV_mean_-LBM | 1.17 (0.36-3.83) | 0.801 |  |  |  |
| TLG-LBM | 1.65 (0.50-5.28) | 0.410 |  |  |  |
| HI-LBM | 3.19 (0.98-10.41) | 0.055 |  | 3.46 (1.05-11.33) | **0.041** |
| Ann Arbor | - | 0.758 |  |  |  |
| I | Reference |  |  |  |  |
| II | 1361.22 (0-5.70*10^88) | 0.943 |  |  |  |
| III | 3979.67 (0-1.65*10^89) | 0.934 |  |  |  |
| IV | 2982.50 (0-1.24*10^89) | 0.937 |  |  |  |
| **OS** |  |  |  |  |  |
| Age | 3.47 (0.70-17.23) | 0.128 |  |  |  |
| Sex | 3.12 (0.38-25.81) | 0.290 |  |  |  |
| BMI | 21.17 (0-8.95*10^10) | 0.787 |  |  |  |
| B symptoms | 7.75 (1.90-31.56) | 0.004 |  | 9.04 (1.89-43.16) | **0.006** |
| Bulky disease | 1.41 (0.28-7.05) | 0.673 |  |  |  |
| Splenomegaly | 0.77 (0.15-3.82) | 0.747 |  |  |  |
| LDH | 2.56 (0.51-12.79) | 0.252 |  |  |  |
| β2-microglobulin | 3.60 (0.85-15.21) | 0.082 |  | 1.66 (0.34-8.06) | 0.528 |
| MIPI score | 2.13 (0.43-10.58) | 0.355 |  |  |  |
| Ki-67 score | 2.44 (0.30-20.22) | 0.407 |  |  |  |
| MTV | 6.26 (0.74-52.85) | 0.092 |  | 0.94 (0.01-99.99) | 0.980 |
| SUV_max_-LBM | 3.94 (0.79-19.77) | 0.096 |  | 4.57 (0.75-28.01) | 0.100 |
| SUV_mean_-LBM | 25.50 (0.005-1.43*10^5) | 0.462 |  |  |  |
| TLG-LBM | 5.91 (0.71-48.96) | 0.099 |  | 1.68 (0.02-152.22) | 0.823 |
| HI-LBM | 2.30 (0.55-9.65) | 0.253 |  |  |  |
| Ann Arbor | - | 0.938 |  |  |  |
| I | Reference |  |  |  |  |
| II | 0.99 (0-2.16*10^189) | 1.000 |  |  |  |
| III | 737.45 (0-1.57*10^188) | 0.976 |  |  |  |
| IV | 1432.18 (0-3.03*10^188) | 0.973 |  |  |  |

*HR* hazard ratio.

**TABLE S4 |** Univariate and multivariate analyses of BSA-related prognostic factors in relation to PFS and OS using the Cox regression model.

|  | Univariate analysis | |  | Multivariate analysis | |
| --- | --- | --- | --- | --- | --- |
|  | HR (95% CI) | *P* value |  | HR (95% CI) | *P* value |
| **PFS** |  |  |  |  |  |
| Age | 2.51 (1.20-5.24) | 0.041 |  | 2.63 (1.26-5.50) | **0.010** |
| Sex | 1.96 (0.83-4.63) | 0.125 |  |  |  |
| BMI | 1.25 (0.64-2.45) | 0.508 |  |  |  |
| B symptoms | 0.86 (0.30-2.46) | 0.780 |  |  |  |
| Bulky disease | 0.75 (0.31-1.80) | 0.514 |  |  |  |
| Splenomegaly | 0.56 (0.25-1.24) | 0.151 |  |  |  |
| LDH | 0.59 (0.18-1.92) | 0.376 |  |  |  |
| β2-microglobulin | 0.90 (0.42-1.93) | 0.786 |  |  |  |
| MIPI score | 1.73 (0.84-3.54) | 0.135 |  |  |  |
| Ki-67 score | 1.74 (0.72-4.21) | 0.217 |  |  |  |
| MTV | 3.24 (0.44-23.78) | 0.248 |  |  |  |
| SUV_max_-BSA | 1.88 (0.45-7.85) | 0.387 |  |  |  |
| SUV_mean_-BSA | 20.64 (0-12775557.30) | 0.656 |  |  |  |
| TLG-BSA | 1.67 (0.69-4.04) | 0.259 |  |  |  |
| HI-BSA | 4.26 (1.02-17.77) | 0.046 |  | 4.54 (1.09-18.96) | **0.038** |
| Ann Arbor | - | 0.758 |  |  |  |
| I | Reference |  |  |  |  |
| II | 1361.22 (0-5.70*10^88) | 0.943 |  |  |  |
| III | 3979.67 (0-1.65*10^89) | 0.934 |  |  |  |
| IV | 2982.50 (0-1.24*10^89) | 0.937 |  |  |  |
| **OS** |  |  |  |  |  |
| Age | 3.47 (0.70-17.23) | 0.128 |  |  |  |
| Sex | 3.12 (0.38-25.81) | 0.290 |  |  |  |
| BMI | 21.17 (0-8.95*10^10) | 0.787 |  |  |  |
| B symptoms | 7.75 (1.90-31.56) | 0.004 |  | 4.97 (1.15-21.52) | **0.032** |
| Bulky disease | 1.41 (0.28-7.05) | 0.673 |  |  |  |
| Splenomegaly | 0.77 (0.15-3.82) | 0.747 |  |  |  |
| LDH | 2.56 (0.51-12.79) | 0.252 |  |  |  |
| β2-microglobulin | 3.60 (0.85-15.21) | 0.082 |  | 1.81 (0.37-8.89) | 0.464 |
| MIPI score | 2.13 (0.43-10.58) | 0.355 |  |  |  |
| Ki-67 score | 2.44 (0.30-20.22) | 0.407 |  |  |  |
| MTV | 6.26 (0.74-52.85) | 0.092 |  | 3.15 (0.32-31.54) | 0.328 |
| SUV_max_-BSA | 3.80 (0.76-19.11) | 0.105 |  |  |  |
| SUV_mean_-BSA | 23.42 (0.001-8.16*10^5) | 0.555 |  |  |  |
| TLG-BSA | 2.73 (0.52-14.45) | 0.237 |  |  |  |
| HI-BSA | 1.46 (0.36-5.83) | 0.596 |  |  |  |
| Ann Arbor | - | 0.938 |  |  |  |
| I | Reference |  |  |  |  |
| II | 0.99 (0-2.16*10^189) | 1.000 |  |  |  |
| III | 737.45 (0-1.57*10^188) | 0.976 |  |  |  |
| IV | 1432.18 (0-3.03*10^188) | 0.973 |  |  |  |


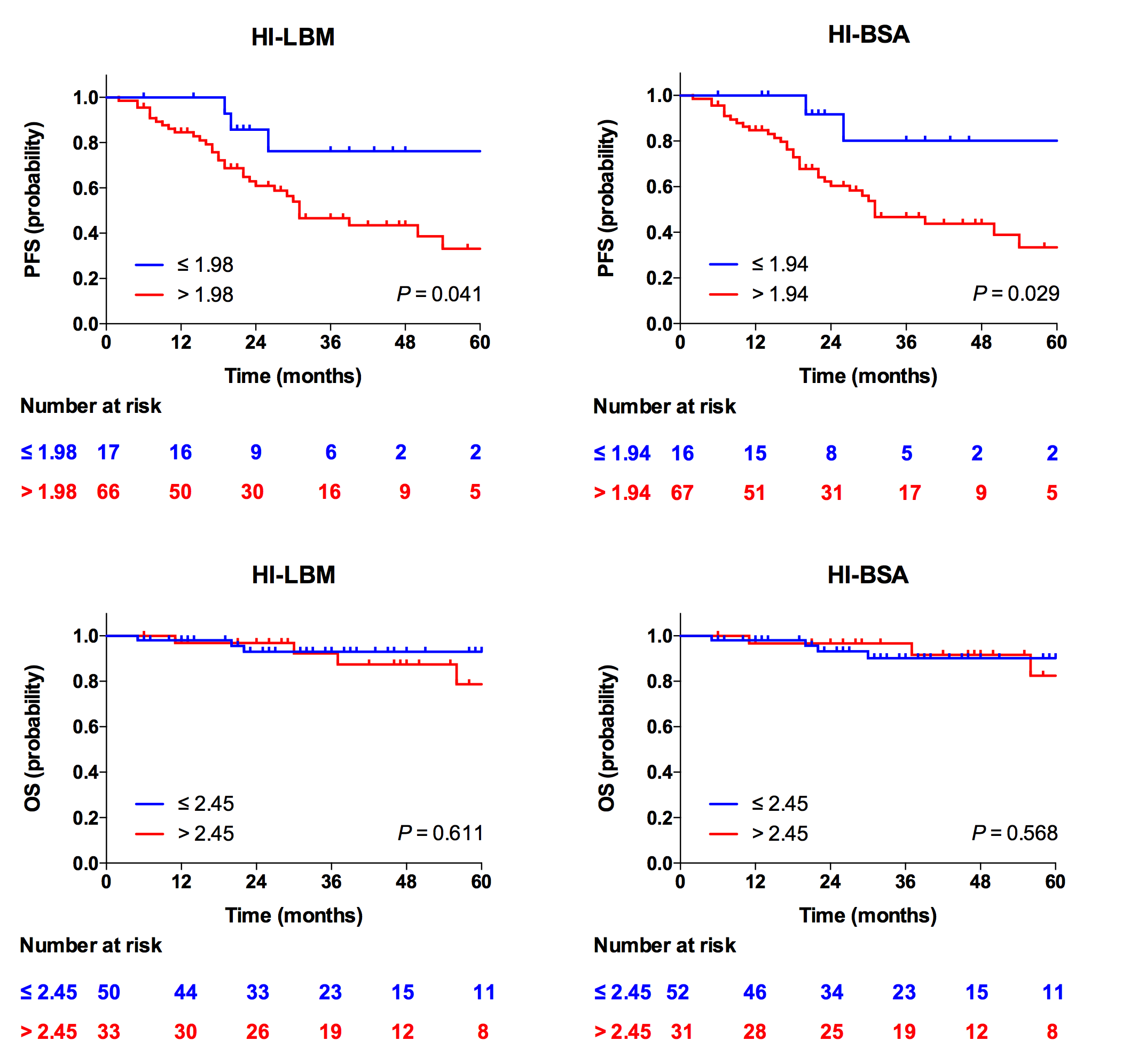


**FIGURE S1** **|** Kaplan-Meier curves for PFS (top row) and OS (bottom row) according to HI-LBM and HI-BSA.
